# Deep fibrous histiocytoma of the index finger: a case report

**DOI:** 10.1080/23320885.2023.2207637

**Published:** 2023-05-05

**Authors:** Hiroki Shibayama, Yuichiro Matsui, Daisuke Kawamura, Daisuke Momma, Takeshi Endo, Yuki Matsui, Yasutaka Yawaka, Kanako C. Hatanaka, Emi Takakuwa, Hirokazu Sugino, Yutaka Hatanaka, Tadashi Hasegawa, Norimasa Iwasaki

**Affiliations:** a Department of Orthopaedic Surgery, KKR Sapporo Medical Center, Sapporo, Hokkaido, Japan; b Department of Orthopaedic Surgery, Faculty of Medicine and Graduate School of Medicine, Hokkaido University, Sapporo, Hokkaido, Japan; c Faculty of Dental Medicine, Hokkaido University, Sapporo, Hokkaido, Japan; d Center for Sports Medicine, Hokkaido University Hospital, Sapporo, Hokkaido, Japan; e Department of Dentistry for Children and Disabled Persons, Graduate School of Dental Medicine, Hokkaido University, Sapporo, Hokkaido, Japan; f Center for Development of Advanced Diagnostics, Hokkaido University Hospital, Sapporo, Hokkaido, Japan; g Department of Surgical Pathology, Hokkaido University Hospital, Sapporo, Hokkaido, Japan; h Research Division of Genome Companion Diagnostics, Hokkaido University Hospital, Sapporo, Hokkaido, Japan; i Department of Surgical Pathlogy, Sapporo Medical University School of Medicine, Sapporo, Hokkaido, Japan

**Keywords:** Deep fibrous histiocytoma, case report, soft tissue tumor in the hand, immunohistochemistry, fusion gene analysis

## Abstract

Our patient presented with an elastic soft mass of his left index finger. Hematoxylin and eosin staining showed a high cellular density with spindle-shaped cells in a storiform pattern. Immunohistochemical staining was positive for CD68, factor XIIIa and α-smooth muscle actin, and negative for CD34, STAT6, S100 protein, and desmin.

## Introduction

Fibrous histiocytomas (FHs) are benign tumors that mostly arise from the skin, which have been referred to as dermatofibromas, sclerosing hemangiomas, and fibrous xanthomas. Approximately 1% of all FHs originate from soft tissues and were previously known as benign fibrous histiocytomas or deep benign fibrous histiocytomas (DBFHs) [1,2], but were renamed deep fibrous histiocytomas (DFHs) according to World Health Organization (WHO) classification of 2013 [3].

DFH is most common in the extremities and the head and neck, and typically presents as a painless, slowly growing mass. Although it is a benign tumor, its local recurrence rate is at least 20%, and distant metastasis may rarely occur [1,2]. In making a pathological diagnosis, immunohistochemistry is important to avoid the misdiagnosis of dermatofibrosarcoma protuberans (DFSP) or solitary fibrous tumors (SFTs) which can occur if only hematoxylin and eosin (HE) staining is used. Cytogenetic abnormalities have been reported in some recent cases [4,5]. Only two reports of DBFH/DFH in the hand have been documented [2,6], and we herein describe a case of DFH in the index finger of a 34-year-old male.

## Report of the case

The patient was a 34-year-old male with no medical or family history of disease. He became aware of a rice grain-sized tumor in his left index finger 8 months previously. Because the tumor gradually grew and he experienced numbness in his finger, he visited a doctor and was referred to our department with a suspected soft tissue tumor.

On physical examination, an elastic, soft mass 1 cm in diameter was observed on the volar and ulnar side of the middle phalangeal area of his left index finger. The overlying skin was normal and there was no redness or local heat. There was no adhesion to the skin, but adhesion to the deep tissue was found. He had mild tenderness and Tinel’s sign was observed. There was mild numbness distal to the tumor and the Semmes–Weinstein monofilament test showed a mild decrease on the ulnar side of the index finger: 3.22 on the ulnar side and 1.65 on the radial side. There was no limitation in the range of motion of his index finger.

**Figure 1.**
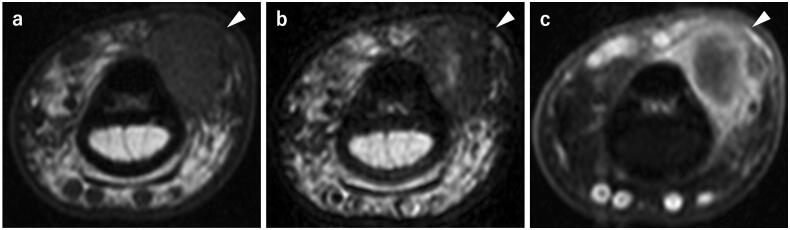
Preoperative magnetic resonance imaging. **a** T1WI; A well-defined mass, 1 cm in diameter, showing iso signal intensity. **b** T2WI; A mass showing low signal intensity with partial internal high signal intensity. **c** Fat-suppressed T1WI-gadlinium showing enhancement on the margin of the mass.

A plain radiograph showed a soft tissue shadow, but there was no abnormality in the bone. Magnetic resonance imaging (MRI) revealed a well-defined mass, 1 cm in diameter, on the volar and ulnar side of the flexor tendon. The mass showed an isotense signal on T1-weighted images (T1WI), a low signal intensity with partial high signal intensity inside the mass on T2-weighted images (T2WI), and contrast enhancement on the margin of the mass was seen with gadolinium (Figure 1(a–c)). Based on these findings, fibroma of the tendon sheath was suspected, and a tenosynovial giant cell tumor and malignant tumors were considered to be a differential diagnosis.

**Figure 2.**
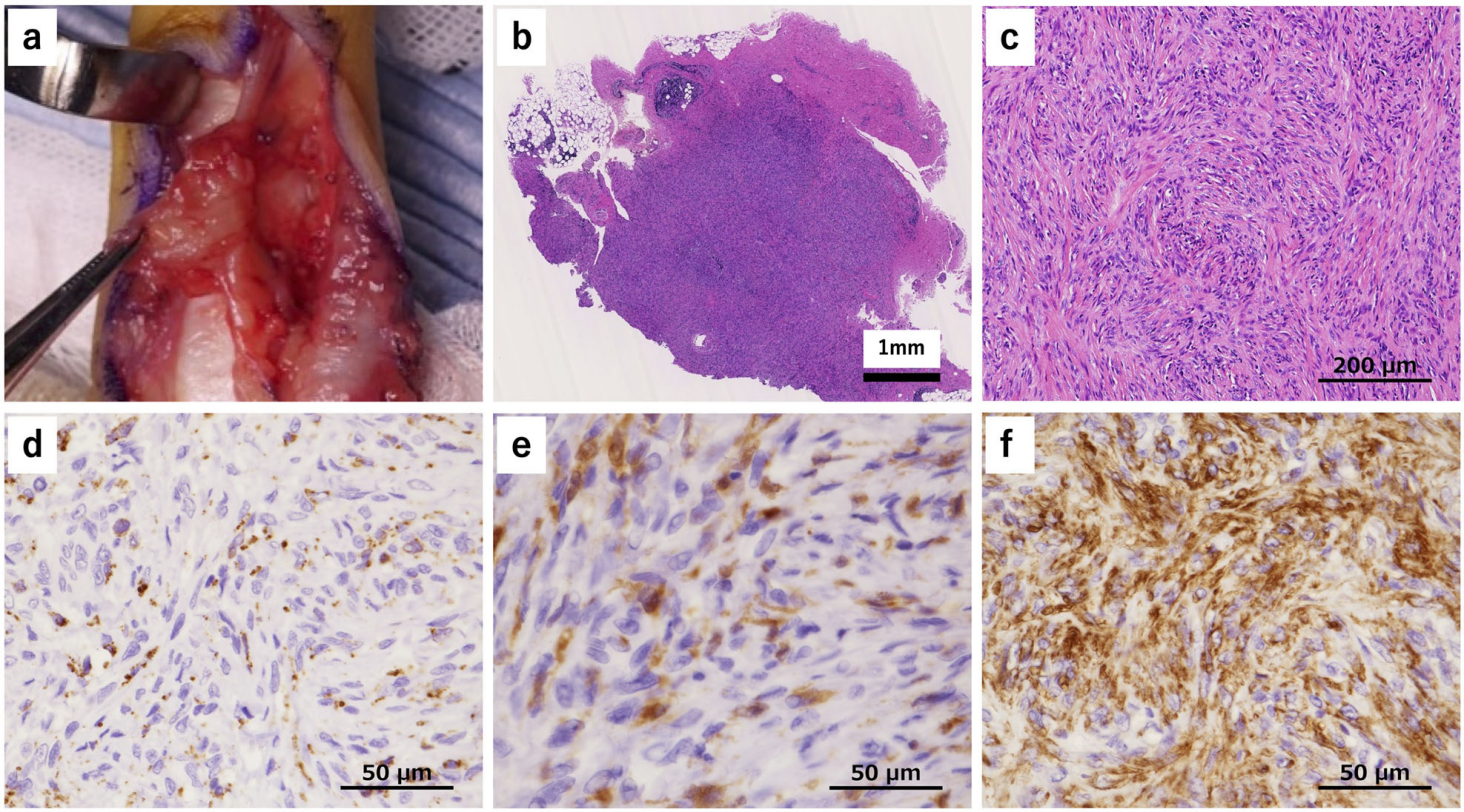
Intraoperative findings and histopathological findings (hematoxylin and eosin staining, and immunohistochemical staining). **a** A yellowish-red mass was detected which was easily dissected from the skin and the tendon sheath. The mass strongly adhered to the neurovascular bundle on the ulnar side. **b** The tumor was relatively well circumscribed. **c** The lesions had high cellular density rather than fibrous tissues, with spindle-shaped cells in a storiform pattern. **d** CD68 was positive in spindle- shaped cells and it presented granular cytoplasmic pattern. **e** Factor XIIIa was also positive in spindle-shaped cells. **f** α-SMA was positive in the majority of spindle-shaped cells.

Because the size of the tumor had increased and numbness and hypesthesia were observed, we performed surgical resection after obtaining informed consent from the patient. After making a Bruner’s skin incision, a yellowish-red mass was detected and it was easily dissected from the skin and the tendon sheath. However, it strongly adhered to the neurovascular bundle on the ulnar side (Figure 2(a)), so required careful dissection and excision. One year after the operation, there was no sign of local recurrence or numbness, and hypesthesia had improved.

### Histopathological findings

HE staining showed that the tumor was relatively well-circumscribed and contained no cutaneous adnexa (Figure 2(b, c)). The lesions had a high cellular density rather than fibrous tissues, with spindle-shaped cells mainly seen in a storiform pattern and partially in a short fascicular pattern. There was no nuclear pleomorphism or hyperchromasia, and no staghorn vessels. The lesions did not have giant cells, hemosiderin, a hemangiopericytoma-like vascular pattern, or a slit-like pattern. Immunohistochemical staining revealed CD68 and factor XIIIa-positive histiocytes (Figure 2(d, e)). CD34 was negative and α-smooth muscle actin (α-SMA) was positive (Figure 2(f)). However, the signal transducer and activator of transcription 6 (STAT6), S100 protein, and desmin were negative. On the basis of these findings, we ruled out fibroma of the tendon sheath, tenosynovial giant cell tumor, DFSP, and SFT, and the diagnosis of DFH was confirmed. Dermatofibroma was ruled out by findings of the normal overlying skin, easy dissection from the skin and no cutaneous adnexa in HE staining. Because DFH rarely occurs in the hand, we performed additional cytogenetic analysis.

### Fusion gene analysis

There have been two reports on genetic abnormalities in DFH, both of which indicate abnormalities in the protein kinase system [4,5]. Plaszczyca et al. reported three fusion genes *(PDPN-PRKCB, LAMTOR1-PRKCD, and CD63-PRKCD)*, which were used for genetic analysis. Although we conducted an RT-PCR assay for three genetic fusions using formalin-fixed paraffin-embedded tissues in accordance with a previously reported method [4], none of them were detected (data not shown). Next, using a 5′/3′ imbalance assay using quantitative PCR [7], we predicted the presence of fusion genes by unbalanced expression between the 5′ and 3′ regions of *PRKCB* and *PRKCD*. However, no differential expression that could be attributed to the oncogenic fusion gene was observed. Based on these results, we concluded that abnormal genetic fusions were unlikely in this case. To rule out DFSP, *COL1A1-PDGFB*, a genetic abnormality invariably found in DFSP, was analyzed with fluorescence *in situ* hybridization and this fusion gene was not detected.

## Discussion

According to the WHO classification of tumors, 5th edition, 2020, DFH is defined as a benign FH arising from soft tissue and rarely metastasizing [8]. About half of all DFHs occur in the extremities, followed by the head and neck, and about 10% of all cases originate from internal organs such as the retroperitoneum and mediastinum [1,2]. Clinically, DFHs often present as a painless, slowly growing mass. They occur in all age groups, are slightly more common in males than females, and almost all cases are treated by marginal resection. Fletcher et al. reported that four (33%) of 12 DFH cases progressed to local recurrence an average of 4.6 years after marginal resection, and that DFH had a high risk of local recurrence [1]. Moreover, Gleason et al. found that about 5% of DFH cases had distant metastasis, and that those with large primary lesions had a higher risk of metastasis than those with smaller lesions [2]. Only two case series have been documented of DBFH/DFH, with zero of 21 cases and one of 69 cases, respectively, occurring in the hand [1,2]. One other case report of DBFH/DFH has been reported in the hand, giving a total of two to date [2,6].

Although no studies on DBFH/DFH imaging have been identified, preoperative MRI, in this case, showed an isotense signal on T1WI, low signal intensity with partial high signal intensity inside the mass on T2WI, and contrast enhancement by gadolinium on the margin of the mass. Benign soft tissue tumors, that frequently occur in the hand, include tenosynovial giant cell tumors, fibromas, lipomas, schwannomas, and hemangiomas [9,10]. Of these tumors, only fibromas show a low signal intensity on T2WI. This indicates that DFH should be included in the differential diagnosis when soft tissue tumors occurring in the hand show a low signal intensity on T2WI.

HE staining of DFH typically shows spindle-shaped cells that are densely arranged in a storiform pattern, usually without nuclear pleomorphism or hyperchromasia [8]. From these characteristics, DFSP, SFT, schwannomas, and leiomyomas can be distinguished as differential diagnoses. Because DFH is not well recognized, misdiagnosis can occur by pathologists who do not specialize in soft tissue tumors [1]. Thus, immunohistochemistry is important for an accurate diagnosis of DFH, and the above diseases can be differentiated by staining for CD68, factor XIIIa, CD34, α-SMA, S100 protein, STAT6, and desmin. In previous reports, there were no detailed descriptions of CD68 and factor XIIIa expression, with Fletcher stating that factor XIIIa was positive only in macrophages and xanthoma cells [1]. In other case reports of DFH, only two references clearly stated both, with CD68 negative and factor XIIIa locally positive, and CD68 and factor XIIIa were positive in xanthoma cells. In other reports, CD68 has been reported to be both negative and positive, and factor XIIIa has been reported to be locally positive. A positive factor XIIIa result was shown to be especially characteristic for DFH [1], which was obtained in the present case. Additionally, HE staining showed a high cellular density rather than fibrous tissues with spindle-shaped cells, and immunohistochemistry staining revealed a large number of CD68 and factor XIIIa-positive histiocytes. Therefore, it was possible to differentiate DFH from the fibroma of the tendon sheath. Generally, HE staining of half of DFH and all DFSP shows variable cell sizes and shapes, an admixture of plasma cells, lymphocytes, histiocytes, including foamy ones, hemosiderin, and collagen trapping at the periphery; all of which are characteristics of dermatofibroma. Although we ruled out dermatofibroma due to the findings of the normal overlying skin, easy dissection from the skin, and lack of cutaneous adnexa in HE staining, focal minor dermal involvement has been reported in some DFH cases [2]. DFSP can be ruled out because the lesion is a small, deep, well-defined lesion clinically, the CD34-positive cells are not diffuse in only a small portion of the lesion, and factor XIIIa-positive cells are also involved, and *COL1A1-PDGFB* was not detected.

Several cytogenetic abnormalities in DFH have recently been reported [4,5]. Here, we investigated the presence of three fusion genes (*PDPN–PRKCB*, *LAMTOR1–PRKCD*, and *CD63–PRKCD*) associated with FH by RT-PCR, but none was detected. We also used an imbalance assay [7] to estimate the presence of fusion genes based on differences in gene expression between 5′ and 3′ regions of *PRKCB* and *PRKCD*; however, no difference in expression that could be attributed to fusion genes was observed. These findings suggest that the previously reported genetic abnormal fusions were unlikely in this case. However, not all cases of DFH show these fusion genes, and most are negative [5], so the absence of genetic abnormalities does not mean that DFH can be ruled out. Indeed, the relationship between DFH and cytogenetic abnormalities remains unclear, and further research is needed.

## Supplementary Material

Supplemental Material
