# Supplementary material for: Deep fibrous histiocytoma of the index finger: a case report
Source: Case Reports Plast Surg Hand Surg. 2023 May 5;10(1):2207637. doi: 10.1080/23320885.2023.2207637 (PMC10165924; doi:10.1080/23320885.2023.2207637)
Supplement: Supplemental Material [file ICRP_A_2207637_SM8214.pdf]

施設名	北大病院
標本番号	S22-90425
患者氏名	Y.R

	プローブ	
☐	CIC-DUX4	CIC(19q13): SO/DUX4(4q35): SG
☐	CIC-FOXO4	CIC(19q13): SG/FOXO4(Xq13): SO
☐	HEY1-NCOA2	HEY1(8q21): SO/NCOA2(8q13): SG
☐	BCR-ABL	BCR(22q11.2):SO/ABL(9q34)
☐	SERPINE1-FOSB	SERPINE1(7q22.1): SO/FOSB(19q13.32): SG
✔	**COL1A1-PDGFB**	

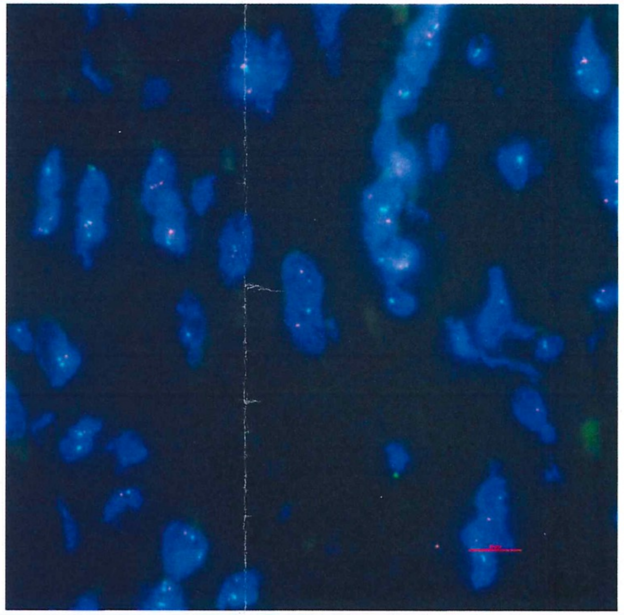

signal pattern	number of cells (n)	(%)
fuson	0	0
negative	50	50
total	50	100

	SO score	SG score
total	100	100
signal / nucleus ratio	2.0	2.0
SO / SG ratio	1.0	
SG / SO ratio	1.0	

解析日：2022.12.9
担当者：青山　智志
確認者：
